# Infant carry sling-associated out-of-hospital cardiac arrest and other circumstances surrounding such events in children under two years: A five-year regional registry analysis

**DOI:** 10.1016/j.resplu.2026.101536

**Published:** 2026-10-21

**Authors:** Andrew Cole, Ollie Zorab, Timothy Godfrey, Sarah Black

**Affiliations:** South Western Ambulance Service NHS Foundation Trust, Abbey Court, Eagle Way, Exeter, Devon EX2 7HY, UK

**Keywords:** Out-of-hospital cardiac arrest, Positional asphyxia, Paediatric resuscitation, Infant

## Abstract

**Introduction:**

Infant carry slings are widely used for infant transport and bonding. However, concerns regarding safety have been raised, including risks of positional asphyxia from improper use, particularly in younger infants. This service evaluation explored the frequency of infant carry sling-associated out-of-hospital cardiac arrest (OHCA) in children under two years old over a five-year period within a regional English ambulance service, placing these findings within the wider epidemiological context.

**Methods:**

This retrospective observational service evaluation used data from an existing OHCA registry and included all patients aged under 2 years who received a resuscitation attempt following cardiac arrest between 1 April 2020 and 31 March 2025. Free-text narratives were reviewed to identify circumstances associated with OHCA, with particular attention paid to carry sling-associated OHCAs.

**Results:**

A total of 162 OHCA incidents in children aged under two years were identified, representing 1.0% of all resuscitation attempts during the five-year period. This included 114 infants aged under one year (excluding neonatal life support patients, *n* = 28). Only two carry sling-associated incidents were identified, both occurring in patients under one year, representing 1.8% of infant OHCAs. The most frequently documented circumstances surrounding OHCA were co-sleeping (33.3%) and patients found unresponsive (e.g. in a cot, bed, sofa or pushchair) (26.3%).

**Conclusions:**

Only two carry sling-associated OHCA incidents were identified over five years, suggesting such events were uncommon within this regional registry. In contrast, other sleep-related circumstances, particularly co-sleeping, accounted for a substantially larger proportion of infant OHCA cases.

## Introduction

Baby wearing describes the practice of carrying an infant or young child in close contact with a caregiver using a sling, wrap or structured carrier.^1^ Baby carry slings comprise a long piece of fabric designed to secure an infant or young child against a caregiver’s body and are commonly used to facilitate physical contact, bonding and breastfeeding.^2^, ^3^ Despite these perceived benefits, concerns have been raised regarding their safety, particularly in young infants. High-profile incidents have highlighted potential for serious harm including positional asphyxia in younger infants with coroner investigation highlighting the absence of clear NHS guidance.^4^

Published evidence concerning baby carry sling related out-of-hospital cardiac arrest (OHCA) is somewhat limited, largely consisting of case reports and small case series.^5^ Reported events occur predominantly in babies less than four months old,^6^ with positional asphyxia identified as a frequent mechanism of death.^7^ OHCA events are commonly reported while an adult caregiver was walking with the infant being carried.^8^ More broadly, infant carrying devices have been associated with traumatic injuries particularly in younger infants, although these data do not specifically address OHCA.^3^, ^5^ In the UK, organisations such as the Lullaby Trust and The Royal Society for the Prevention of Accidents (RoSPA) have highlighted potential risks including positional asphyxia associated with baby carrying devices.^9^, ^10^ Advice and guidance on the safe practice of babywearing is available^11^ however distribution of this information at the point of purchase is still lacking.^12^

Within a regional ambulance service in the South West of England, senior healthcare leaders have raised concerns regarding a perceived increase in carry sling-associated OHCA. However, the frequency and characteristics of these events within the wider population of OHCA in young children have not been previously described. This service evaluation aimed to: (1) determine the frequency of identified carry sling associated OHCA among children under 2 years old, attended by a regional ambulance service; (2) describe temporal patterns in carry sling-associated OHCA over a five-year period; (3) describe the reported circumstances and presumed aetiology of OHCA in children under two years, placing carry sling-associated events within the wider epidemiological context.

## Methods

### Setting

This retrospective observational service evaluation was conducted using data from a regional ambulance service’s OHCA registry in the South West of England. The service contributes data to the UK Out-of-Hospital Cardiac Arrest Outcomes (OHCAO) project.^13^ The OHCAO project provides a national framework for the collection and reporting of prehospital OHCA data across participating UK ambulance services.

The participating ambulance service provides healthcare to a population of approximately 5.5 million people with seasonal influxes of over 23 million visitors annually and covers approximately 10,000 square miles spanning urban, rural and coastal areas. The service provides emergency ambulance response to a variety of medical and traumatic emergencies including OHCA.

### Population

Inclusion criteria comprised all OHCA resuscitation attempts in patients less than two years old between April 1, 2020 and 31 March 2025 (Fig. 1). Neonatal life support (NLS) patients were identified and excluded from further analysis in accordance with recent Utstein reporting guidance.^14^ NLS refers to neonatal resuscitation provided immediately following birth and represents a distinct clinical population and resuscitation context. NLS incidents are included in Table 1 to provide wider epidemiological context.

**Fig. 1:**
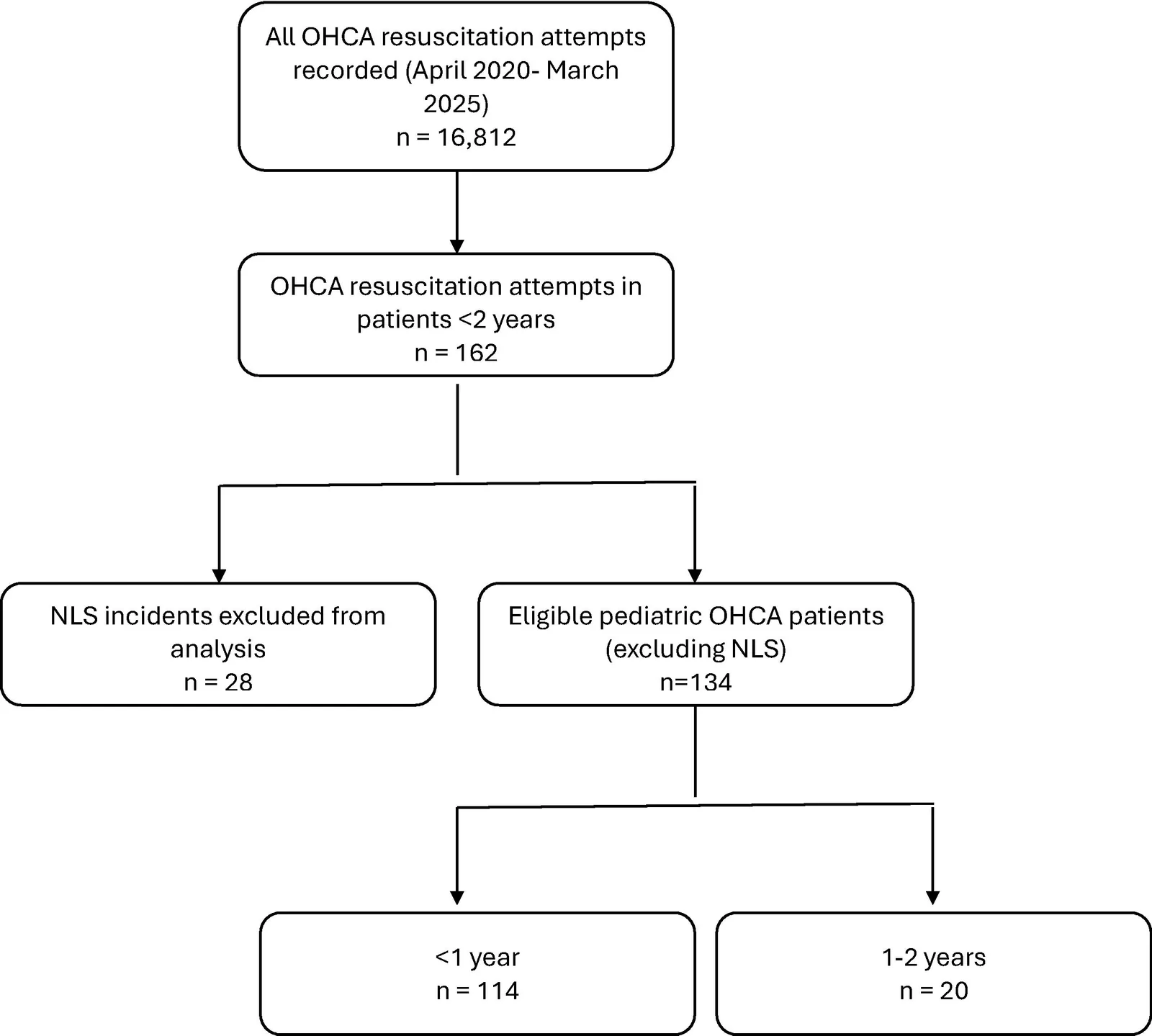
**Flow diagram of OHCA case identification and cohort selection**.

**Table 1:** Annual frequency of OHCA resuscitation attempts in patients under 2 years (2020–2025).

**Registry year**	Newborn life support (NLS)*	**<1year (excl. NLS)**	**1**–**2 years**	**Total resus attempts** **(all ages)**	**Under 2 years % of total resus attempts**
2020–21	8	23	3	3152	1.1%
2021–22	2	33	3	3564	1.1%
2022–23	7	18	7	3590	0.9%
2023–24	6	16	4	3197	0.8%
2024–25	5	24	3	3309	1.0%

Total	28	114	20	16,812	1.0%
Yearly mean	5.6	22.8	4	3362.4	1.0%

* NLS incidents are presented for epidemiological context but have been excluded form subsequent analysis of data for <1 year patients.

### Data collection

Eligible patients and relevant data fields were identified and extracted from the ambulance service’s OHCA registry, including available free-text clinical narratives. Due to the focused nature of this evaluation, only patient age was collected as a demographic variable. All patient-identifiable information was removed prior to analysis.

### Data analysis

Presumed cause was defined as the most likely aetiology of OHCA recorded by the attending clinician(s) within the original source data and was recorded using recommended Utstein classifications.^14^ Within the ‘medical’ classification, the Utstein framework permits additional subgroups, including sudden unexpected infant death (SUID). SUID was not routinely recorded within the source OHCA registry and was therefore not used as a separate classification in this evaluation. Consequently, incidents reporting a presumed medical cause may represent a range of clinical presentations, including where no specific underlying cause was apparent at the time of prehospital assessment.

To supplement the presumed cause classification, which may lack detail regarding the circumstances surrounding an arrest, free-text clinical narratives were reviewed to identify the reported circumstances associated with each OHCA. This was performed by a single reviewer (AC). Circumstance classification was developed iteratively during the review process, reflecting recurring patterns within the dataset. Following initial analysis, summary data were presented and reviewed with subject matter experts (OZ and TG), to identify any potentially anomalous findings and to support interpretation of the results. Where multiple circumstances were documented, the circumstance considered most clinically relevant to the subsequent OHCA was assigned based on the overall clinical narrative, with no predefined hierarchical order applied. Missing or insufficient data required to categorise reported circumstances were labelled as ‘data missing’.

Descriptive statistical analysis was performed using Microsoft Excel (Version 16.105.3), continuous variables are presented using summary statistics, whilst categorical data are presented as frequencies and percentages. Summary tables and graphs are used to present findings.

### Ethical considerations

This service evaluation was reviewed and approved by the participating ambulance service’s Research and Audit department and supported by senior clinical leaders who commissioned the project. It involved secondary analysis of routinely collected clinical data, with no direct patient involvement or intervention. Data extraction was undertaken by an individual with authorised access to the OHCA registry as part of their professional role (AC). All data were anonymised prior to analysis. In accordance with the Health Research Authority guidance, ethical approval was not required as the project was classified as a service evaluation.

## Results

Among 16,812 OHCA resuscitation attempts between 2020 and 2025, 162 involved children under 2 years of age, representing 1.0% of all resuscitation attempts (Table 1). Of these, 114 occurred in infants aged less than one year (excluding NLS patients, *n* = 28) and 20 occurred in children aged 1–2 years.

### Carry sling-associated arrests

Two incidents in which the infant was reported to be in a baby carry sling at the time of OHCA were identified during the five-year period. Both patients were under 1 year old representing 1.8% of the 114 patients in this age group. Due to very small number of incidents, meaningful assessment of temporal trends was not possible. Further demographic, clinical and incident-specific details were not presented, as inclusion could increase the risk of patient identification and compromise confidentiality due to low patient numbers.

### Presumed cause and reported circumstances associated with OHCA

Presumed cause and reported circumstances associated with OHCA are presented separately in Table 2. ‘Medical’ was the most recorded presumed cause in both age groups. Due to low annual incidence of OHCA in this cohort, data were combined across the five-year period for analysis. Fig. 2 shows absolute frequency of reported circumstances leading to OHCA in infants aged less than 1 year (excluding NLS). The five patients categorised as ‘witnessed collapse – no other circumstance’ refer to incidents where a caregiver witnessed the collapse in the absence of any other clear circumstance identified (e.g. recently unwell or aspiration while feeding).

**Table 2:** Presumed aetiology and reported circumstances of OHCA in patients under 2 years.

Aetiology*	**<1 year (excl NLS) (*n*)**	**% of total <1 year**	**1**–**2 years (*n*)**	**% of total 1**–**2 years**	**Combined total (*n*)**	**% of combined total**
Medical	86	75.4%	10	50.0%	96	71.6%
Asphyxia	18	15.8%	1	5.0%	19	14.2%
Drowning	3	2.6%	6	30.0%	9	6.7%
Trauma	3	2.6%	3	15.0%	6	4.5%
Other	4	3.5%	0	0.0%	4	3.0%
Total	114		20		134	

**Reported circumstance leading to OHCA**+						
Co-sleeping	38	33.3%	0	0.0%	38	28.4%
Found unresponsive (in cot/bed/sofa)	30	26.3%	4	20.0%	34	25.4%
Recently unwell (e.g. febrile illness, respiratory symptoms, poor feeding)	15	13.2%	5	25.0%	20	14.9%
Known complex medical history	9	7.9%	1	5.0%	10	7.5%
Witnessed collapse	5	4.4%	0	0.0%	5	3.7%
Data missing	4	3.5%	0	0.0%	4	3.0%
Drowning/submersion	3	2.6%	6	30.0%	9	6.7%
Trauma	3	2.6%	3	15.0%	6	4.5%
Choking/asphyxia	3	2.6%	1	5.0%	4	3.0%
Arrest following hospital discharge	2	1.8%	0	0.0%	2	1.5%
In carry sling/baby carrier	2	1.8%	0	0.0%	2	1.5%

* Aetiology was based on presumed cause of OHCA recorded by the attending clinician(s) as recorded within the registry and does not represent a confirmed pathological or coronial cause.

+ Reported circumstance leading to OHCA were identified from registry free-text clinical narratives and represent the documented circumstances at the time of the incident. Some circumstances may overlap conceptually with presumed aetiology (e.g. drowning and trauma). However, these variables were considered separately, with reported circumstances reflecting the documented context of the event rather than confirmed underlying cause. Where multiple circumstances were documented, a single primary circumstance was assigned.

**Fig. 2:**
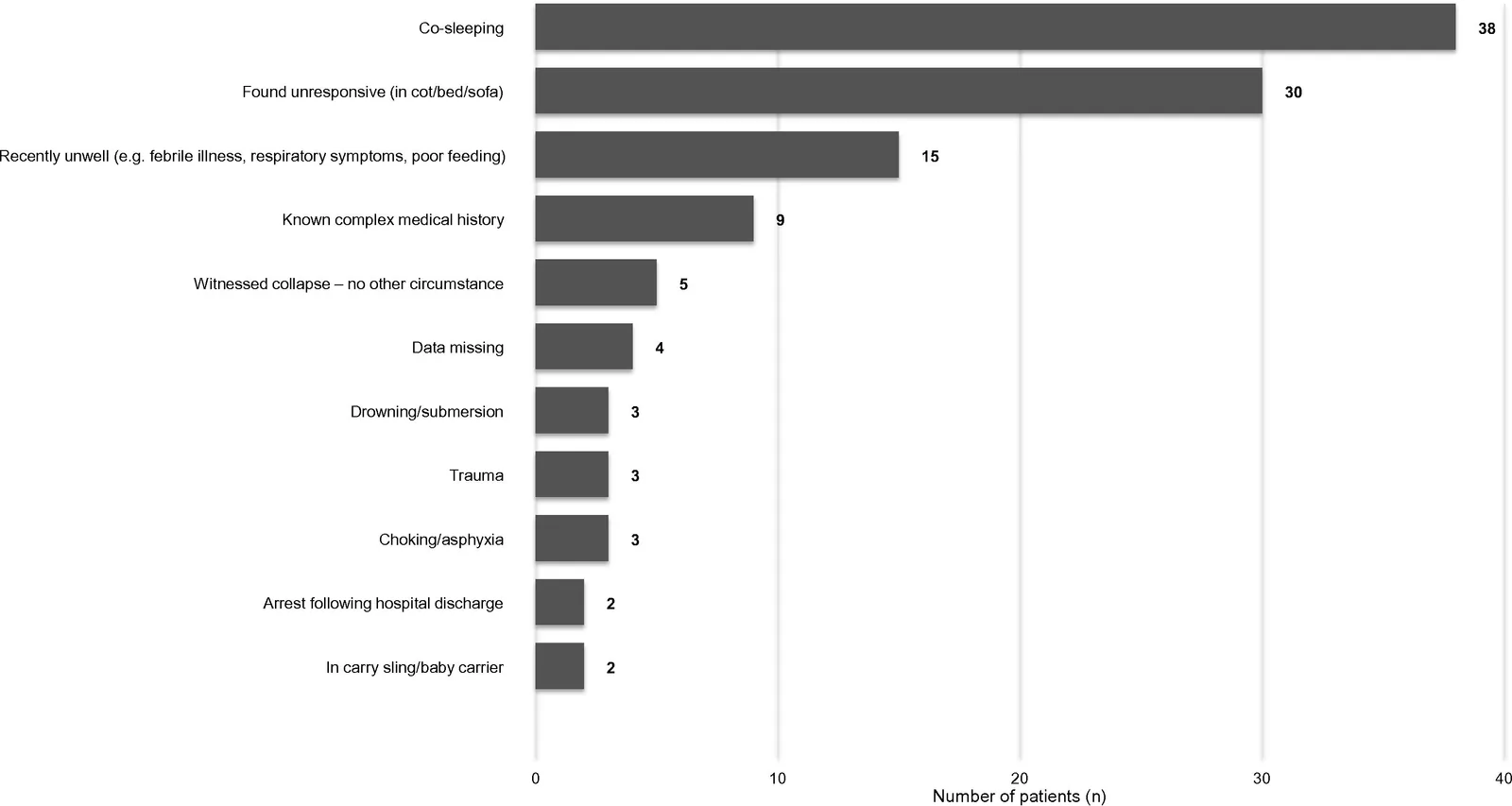
**Reported circumstances associated with OHCA in infants aged <1 year, excluding NLS**.

## Discussion

This service evaluation was developed in response to concerns regarding infant OHCA associated with carry sling use. Over a five-year period, two carry sling related OHCAs were identified among 114 OHCA patients aged less than 1 year (excluding NLS), representing 1.8% of this cohort. Both infants were in ROSC at arrival to the emergency department, but neither survived to 30 days. Given the small number of identified patients, these findings should be interpreted cautiously and do not permit assessment of temporal trends or estimation of the wider burden of carry sling related harm.

### Carry sling-associated OHCA: observations and limitations

Although carry sling use has been associated with positional asphyxia and serious infant injury, evidence specifically relating to carry sling associated OHCA remains extremely limited and consists predominantly of case reports,^7^ case series,^8^ safety alerts^11^ and coronial investigations.^4^ The two cases identified in this evaluation therefore provide additional regional data describing the occurrence of OHCA in association with carry sling use. However, the registry data do not permit definitive determination of the underlying mechanism of arrest nor establish a causal relationship between carry sling use and OHCA.

Organisations such as the Lullaby Trust and The Royal Society for the Prevention of Accidents (RoSPA) have highlighted risks associated with carry sling use and following research, have developed educational resources and guidelines to promote correct and babywearing practices.^9^, ^10^ The ‘TICKS’ acronym (Tight, In view at all times, Close enough to kiss, Keep chin off chest, Supported back) provides guidance on appropriate positioning when using a carry sling.^11^ However, the extent to which such guidance has been adopted and its impact on safety outcomes remain unclear. Recently published survey data suggested that a large proportion of babywearing parents were aware of ‘TICKS’, however, advice and guidance on the safe practice of babywearing at the point of carry device purchase remain limited.^12^

### Carry sling OHCA in the broader context

Over the reporting period, 162 OHCA incidents were identified in patients under 2 years old. These comprise three groups: NLS, comprising 17.3% (*n* = 28); Infants <1 year (excluding NLS), comprising 70.4% (*n* = 114); and children aged 1–2 years, comprising 12.3% (*n* = 20).

Within the <1 year (excl. NLS) group, the most frequently reported circumstance was co-sleeping, presenting in 33.3% of incidents (*n* = 38), which is broadly consistent with national child mortality data. While these populations are not directly comparable, the National Child Mortality Database reported that 52% of unexplained infant deaths occurred while sharing a sleeping surface with 92% occurring in hazardous situations (e.g. with an adult who had consumed alcohol or on a sofa).^15^

The second most frequent circumstance in this evaluation involved infants found unresponsive in locations such as cots, beds, sofas or pushchairs (26.3%). Together, these findings demonstrate that sleep-related circumstances were substantially more common than carry sling-associated OHCA within this cohort and provide broader context for interpreting the two identified carry sling-associated patients. This also reinforces the importance of safe sleep practices and established public-health risk reduction measures.^16^

### Limitations

This service evaluation was not without limitations. While carry sling-associated OHCA appear uncommon within this dataset, this evaluation does not capture other potential harms associated with carry sling use, such as trauma sustained from falls where OHCA did not occur, or infants who may be in respiratory distress or who become critically unwell without subsequent OHCA. The low number of carry sling-associated incidents limits any conclusion that can be drawn regarding frequency, characteristics or temporal patterns.

Reported circumstances associated with OHCA were inferred from available clinical documentation and may have been informed by collateral history provided by caregivers or other witnesses. These circumstances have not received the scrutiny of a coronial process. Similarly, the registry records presumed cause as assessed by attending clinician(s) in the prehospital setting and does not contain subsequent hospital or coronial findings. Neither the reported circumstances nor the registry recorded presumed cause can be interpreted as a confirmed underlying pathological or coronial cause of OHCA. Causal relationships between reported circumstances and OHCA therefore cannot be established.

The OHCA registry is a population-level dataset designed for broad data capture across all age groups. Detailed data were only collected for resuscitation attempts, with only limited detail available for those found deceased on arrival where resuscitation was not attempted. As a result, some OHCA incidents in under 2-year-olds where resuscitation was not attempted may not have been captured within the detailed registry dataset and could therefore have been missed from this analysis. This evaluation is also subject to the limitations inherent in retrospective registry studies. Classification of reported circumstances associated with OHCA relied on documented information within clinical records and free-text narratives and was undertaken by a single reviewer. This may have led to misclassification in some instances where documentation was incomplete, ambiguous or where inconsistent terminology was used. The findings may not be directly generalisable to other ambulance services or geographical regions, where population characteristics, service configuration and clinical practice may differ.

The evaluation period encompassed the COVID-19 pandemic, during which changes in household behaviours, childcare arrangements and social contact patterns may have altered the incidence and circumstances of infant OHCA events, for example, the potential for less public baby carry sling use. Consequently, observed case numbers may not be fully representative of pre and post pandemic patterns.

## Conclusion

Although concerns regarding carry sling-associated OHCA have prompted safety alerts and coronial investigations, only two incidents were identified over the five-year period. In contrast, sleep-related circumstances, particularly co-sleeping, were commonly reported among infant OHCA cases. Within this regional registry, carry sling-associated OHCA was uncommon and represented a small proportion of identified infant OHCA patients. However, the small number of identified cases limits assessment of temporal trends and highlights the need for larger datasets to further characterise the epidemiology of these events.

## Declaration of generative AI and AI-assisted technologies in the manuscript preparation process

During the preparation of this work the authors used ChatGPT (Open AI) in order to improve clarity and flow of the written manuscript while maintaining brevity. No form of artificial intelligence was used in data collection, data analysis or interpretation of results, nor was any patient identifiable information ever provided to any AI software. After using this tool/service, the authors reviewed and edited the content as needed and take full responsibility for the content of the published article.

## Funding sources

This work was supported by South Western Ambulance Service NHS Foundation Trust. This service evaluation did not receive any specific grant from funding agencies in the public, commercial or not-for-profit sectors.

## Data statement

Data generated and analysed during this service evaluation are not publicly available. The collected data contain sensitive and confidential information that could potentially identify individual participants and/or organisational information which could compromise participant confidentiality and organisational privacy if shared. The data are stored securely by SWASFT in accordance with the organisation’s data governance policies and are not available for public sharing.

## CRediT authorship contribution statement

**Andrew Cole:** Writing – review & editing, Writing – original draft, Project administration, Methodology, Investigation, Formal analysis, Data curation, Conceptualization. **Ollie Zorab:** Writing – review & editing, Supervision, Methodology, Conceptualization. **Timothy Godfrey:** Writing – review & editing, Supervision, Methodology, Conceptualization. **Sarah Black:** Writing – review & editing, Supervision.

## Declaration of competing interest

All authors are employed by the ambulance service in which this service evaluation was conducted. The authors declare that they have no known competing financial or personal interests related to this work.
